# Ten Years After SINS: Role of Surgery and Radiotherapy in the Management of Patients With Vertebral Metastases

**DOI:** 10.3389/fonc.2022.802595

**Published:** 2022-01-27

**Authors:** Nicolas Serratrice, Joe Faddoul, Bilal Tarabay, Christian Attieh, Moussa A. Chalah, Samar S. Ayache, Georges N. Abi Lahoud

**Affiliations:** ^1^ Institut de la Colonne Vertébrale et des NeuroSciences (ICVNS) - CMC Bizet, Paris, France; ^2^ Department of Neurosurgery, Centre Hospitalier de la Côte Basque, Bayonne, France; ^3^ Univ Paris Est Créteil, Excitabilité Nerveuse et Thérapeutique (ENT), EA 4391, Créteil, France; ^4^ Assistance Publique - Hôpitaux de Paris (AP-HP), Henri Mondor University Hospital, Department of Clinical Neurophysiology, DMU FIxIT, Créteil, France

**Keywords:** spinal metastases, cancer, spinal cord compression, surgery, radiotherapy, spine instability neoplastic score (SINS)

## Abstract

The objective of the different types of treatments for a spinal metastasis is to provide the best oncological and functional result with the least aggressive side effects. Initially created in 2010 to help clinicians in the management of vertebral metastases, the Spine Instability Neoplastic Score (SINS) has quickly found its place in the decision making and the treatment of patients with metastatic spinal disease. Here we conduct a review of the literature describing the different changes that occurred with the SINS score in the last ten years. After a brief presentation of the spinal metastases’ distribution, with or without spinal cord compression, we present the utility of SINS in the radiological diagnosis and extension of the disease, in addition to its limits, especially for scores ranging between 7 and 12. We take this opportunity to expose the latest advances in surgery and radiotherapy concerning spinal metastases, as well as in palliative care and pain control. We also discuss the reliability of SINS amongst radiologists, radiation oncologists, spine surgeons and spine surgery trainees. Finally, we will present the new SINS-derived predictive scores, biomarkers and artificial intelligence algorithms that allow a multidisciplinary approach for the management of spinal metastases.

## Introduction

The spine is the most common site of bone metastases in general and skeletal metastases in particular, with a prevalence of 30-70% and 20-40% in cancer patients respectively  (1, 2).Thus, spinal metastases are a concerning health issue as well as an economic burden  (3, 4), despite the fact that the management of these patients has considerably evolved both in terms of surgery and radiotherapy (RT)  (5–8).

Ten years ago, the Spine Instability Neoplastic Score (SINS) introduced by the Spine Oncology Study Group (SOSG), quickly established itself as a reliable and predictive tool for clinicians allowing them to decide whether patients with spinal neoplastic disease of the spine would benefit from surgical intervention  (9, 10). Indeed, it assesses and scores 6 variables (**Table 1**): location of the lesion, characteristics of pain, type of bony lesion, radiographic spinal alignment, degree of vertebral body destruction, and involvement of posterolateral spinal elements. The scores for each variable are added, and a final score is obtained. The minimum score is 0, and the maximum score is 18. A score of 0 to 6 denotes stability, a score of 7 to 12 denotes indeterminate (possibly impending) instability, and a score of 13 to 18 denotes instability. For scores greater than 7, a surgical consultation is recommended  (9). Despite the straightforward management choices for scores less than 7 and greater than 12, decision-making becomes challenging for the majority of patients who have scores between 7 and 12 with lesions considered as “potentially unstable”.

**Table 1:** SINS classification.

**Location:** junctional: occiput-C2, C7-T2, T11-L1, L5-S1 mobile spine: C3-C6, L2-L4 semirigid: T3-T10 rigid: S2-S5	3 points 2 points 1 point 0 point
**Pain:** mechanical pain: improves with recumbency or pain with movement or spinal loading occasional pain but not mechanical painless lesion	3 points 1 point 0 point
**Bone lesion:** lytic mixed blastic	2 points 1 point 0 point
**Radiographic spinal alignment:** subluxation/translation de novo deformity (kyphosis/scoliosis) normal alignment	4 points 2 points 0 point
**Vertebral body collapse:** >50% collapse <50% collapse no collapse with >50% vertebral body involved none of the above	3 points 2 points 1 point 0 point
**Posterior spinal element involvement:** bilateral unilateral none of the above	3 points 1 point 0 point

In this review, we describe the evolution of our practice over the past ten years (*i.e*., since the publication of the SINS), and we provide the latest updates in the management of vertebral metastases.

## Review

### Primary/Secondary Spinal Tumors

Since advanced cancers frequently metastasize to the vertebral body as part of the musculoskeletal system  (11, 12), we decided to mainly focus on secondary spinal tumors with or without a known primitive tumor. Furthermore, we will discuss briefly, later in this manuscript, primary vertebral tumors with multiple level spreading, such as myeloma  (13), and we will present the specificities of spinal metastases encountered at the cranio-cervical junction.

### Distribution of Spinal Metastases

Predilection of spinal metastases is not completely understood. Metastases are located at a single spinal level in 70% of the cases, with a predilection to the thoracic spine, followed by the lumbar, the cervical, and the sacral levels  (1, 14). Metastases involving more than two spinal sites were found in 30% of patients. Vertebral body is more frequently involved in metastatic disease compared to the posterior elements. One third of patients presented a circumferential spine involvement (body and posterior elements). An associated epidural compression was present in half of the patients  (14).

Bone is one of the preferential sites of distant metastases from malignant tumors, with the highest prevalence observed in breast and prostate cancers. Even if some tumors have a significant propensity to localize at certain vertebral levels, it remains impossible to conclude on a specific tumor metastatic profile  (14). Primitive neck tumors, such as ENT and thyroid tumors, spread towards the cervical spine whereas pelvic tumors (especially bladder and prostate) metastasize to the lumbar spine. All tumors present a tropism for thoracic vertebrae. Interestingly, significant tumor/vertebrae associations were identified: lung and thyroid for L1, bladder for L5, breast for C6, prostate for L1-L4, multiple myelomas for C7, T3-T7 and L1-L4  (14).

### Survival

**Table 2** presents the overall survival for the main cancers with spinal metastases. Survival estimation in patients with spinal metastases can significantly influence treatment recommendations. Survival was generally overestimated by oncologists and spine surgeons and longer estimated survival seemed to lead to more invasive procedures  (22, 23).

**Table 2:** Overall survival for different cancer with spinal metastases.

		Median overall survival time (months)
Thyroid cancer with spinal metastases	(15*)	9.1 years
Multiple myeloma with spinal metastases	(16*)	108 months
Kidney cancer with spinal metastases	(17)	100 months
Breast cancer with spinal metastases	(18*)	43.9 months
Prostate cancer with spinal metastases	(19*)	28.8 months
Lung cancer with spinal metastases	(20*)	5.9 months
Colorectal cancer with spinal metastases	(21)	5 months

*French national multi-center database.

### Metastases Associated With Spinal Cord Compression

Spinal cord compression secondary to epidural metastases can be present in 5 to 20% of cases, and is associated with neurological impairment, decreased mobility and worse quality of life  (1, 2). The mean survival was 12.3 months  (24). At this point, treating such patients with metastatic epidural spinal cord compression remains a challenge, and a multidisciplinary approach is highly recommended. Hence, the main goal of the treatment is to avoid spinal cord or *cauda equina* compression, which negatively affects prognosis and functional recovery.

### An Early Diagnosis for a Better Care

The average time between symptoms onset and diagnosis is 4 months (25). The main symptom is pain (revealing the disease in 1/3 of patients), which increases morbidity and reduces quality of life  (6). At the thoracic level, the first manifestation is often paraparesis. It is important to ensure the absence of associated sphincter disorders. Making an early diagnosis before neurological deficits is of utmost importance, in order to avoid irreversible lesions. With the widespread use of magnetic resonance imagery (MRI) and easier access to diagnostic tools, any patient with cancer presenting with back pain should be immediately investigated  (5, 26) and in case of neurological deficit, decompressive surgery should be urgently realized  (27).

### SINS and Radiology

With five out of six variables being radiological, a full spine computed tomography (CT) scan and a pan-medullary MRI are essential in calculating SINS.

A full spine CT, in addition to precisely locating lesions, provides a good spatial resolution compared to classical X-rays. It permits a fine analysis of the trabecular meshwork, and a thorough study of the vertebra  (28, 29). SINS variables such as the type of bony lesion, the radiographic spinal alignment, and the degree of vertebral body destruction are mandatory. However, CT-scans are limited in identifying epidural infiltration and spinal cord impact (29).

A pan-medullary MRI is also essential for localizing multiple bone lesions, assessing dural or foraminal epidural invasion, posterior wall involvement, deformity, or sometimes severe compression of neurological elements  (28). Involvement of posterolateral spinal elements is an important variable of the SINS. In the latter situation, a hyperintensity T2 signal of the spinal cord reflects edema or gliosis and is considered a severity criterion  (30).

As stated above, SINS is a reliable tool for radiologists to evaluate tumor-related spinal instability. It accurately differentiates between stable, potentially unstable and definitely unstable lesions and, therefore, can guide the need for surgical consultation  (26).

### Extension of the Disease

In the absence of a known primitive tumor, an assessment with a thoraco-abdomino-pelvic CT-scan should be systematically carried out. Depending on the tumor origin, a prior embolization may be performed, especially in the context of kidney and thyroid tumors, melanomas and choriocarcinomas  (31). Screening for brain disease extension is also very important  (32), especially that postoperative complications are increased in case of brain metastases.

### A Multidisciplinary Approach

Management of spinal metastases warrants a multidisciplinary approach, *i.e.*, including medical oncology, radiation oncology, and surgical consultation  (33, 34). This approach aims primarily to relieve pain and improve quality of life by adapting a case specific strategy in order to ensure the best outcome and the lowest risk. This strategy allows a multidimensional evaluation by adapting a patients’ tailored treatment in terms of pain management, daily life improvement, and hospitalization duration. In this context, the decision to decompress the neurological elements and/or secure the mechanical stability cannot be solely based on SINS. A thorough assessment should be undertaken by including the number of the lesions, their spinal localizations, the existence of potential neurological damage/threat or a potential mechanical instability, their response to radio and/or chemotherapy, their primary origin and the previous treatments. In addition, patients’ characteristics should be taken into consideration by examining the level and intensity of pain, the only clinical variable included in the SINS. Other scales/scores as Visual Analogic Scale (VAS)  (35), World Health Organization (WHO) scale  (36), Karnofsky Performance Status (KPS), functional scale  (37), Tokuhashi  (38), life expectancy scale  (39), malnutrition  (40), American Society of Anesthesiologists (ASA) score and ability to withstand treatment  (41–43) can also be useful and complementary to SINS.

### What About Scores Ranging Between 7 and 12

Currently, for scores between 7 and 12, the prognostic value of the SINS is still controversial  (44). Many studies have examined the subgroup of patients in this grey zone who required surgical fixation after an initial conservative approach. Based on these reports, it has been possible to define a cutoff where the spine is considered possibly unstable and would be more likely to require stabilization. This is the case of patients with SINS score of 11 or greater  (45).

### Advances in Surgery

Surgery remains a valuable weapon in the therapeutic arsenal  (46). Surgery for spinal metastases has three main objectives: management of pain, achievement of mechanical stability and preservation or restoration of neurological function. A variety of surgical approaches are available and depend on the tumor location, presence of instability, neurological status, oncological prognosis, general performance status and subsequent treatment measures. In this context, SINS can be used as a crucial clinical tool for surgery, helping clinicians to determine whether instability is present and when surgery would be indicated  (47). Treatment decisions for patients with spinal metastases can be challenging and greatly depends on survival prognosis. Moreover, other parameters such as spinal cord compression and extent of disease dictate whether surgery is the most appropriate option  (48). A wide range of fusion techniques exists, each one tailored to the location of the lesion and the surgery goals. To optimize results, expert knowledge on the techniques and patient selection is of utmost importance.

The advent of new technologies and minimally invasive surgical techniques has helped optimize the treatment of spinal metastases, especially in patients with low performance status. Surgery has become less aggressive, and the use of stereotactic radiosurgery (SRS) has led to a new treatment concept known as “separation surgery”  (49), relying on making a safe space, free of tumor, around the spinal cord or *cauda equina*, and then applying high dose hypofractionated SRS (24-30 Gy in 3 fractions). Hence, separation surgery can provide a 2- to 3-mm safe separation of tumor and spinal cord to avoid radiation-induced damage to the spinal cord. Targets for separation surgery include decompression of metastatic epidural spinal cord compression and spinal stabilization without partial or en bloc tumor resection  (50). This results in less radiation dose, thus protects the neurological elements from the radiation toxicity, and diminishes radiation-induced vertebral fracture which is considered the most serious and prevalent side effect of SRS. Major vertebral resections, especially in radioresistant metastases, are no more needed, and thus complications of surgery are minimized. Laufer et al. showed that postoperative adjuvant SRS following epidural spinal cord decompression and instrumentation is a safe and effective strategy for establishing durable local tumor control regardless of tumor histology-specific radiosensitivity  (49). This less-invasive alternative to radical spinal oncological resection appears to be effective regardless of tumor histology and does not jeopardize the radiographic or clinical response durability  (51). As an example, Spine stereotactic body radiation therapy (SBRT), a derivative of SRS, yields high rates of local tumor control in patients with renal cell cancer, a tumor classically known as radioresistant.

The presence of an osteolytic lesion is more significant than the involvement of the whole vertebral body in predicting the rate of vertebral compression fractures (VCF) after SRS. It has been shown that considerable vertebral destruction (more than 60%) is associated with high risk of VCF or spinal deformity  (52). Several factors associated with survival after spinal SRS for renal cell carcinoma metastases were identified, including local progression, time between first metastasis and primary renal cell carcinoma diagnosis, KPS score, presence of neurological deficits, and progressive metastatic disease. These factors should be taken into consideration when considering a patient for spinal SRS for renal cell carcinoma metastases  (53).

Finally, “Time is Spine”  (5). The best neurological prognosis after surgery for metastatic epidural spinal cord compression is obtained when surgery is done in the 48 hours following the installation of the neurological signs and symptoms  (54). Moreover, the postoperative outcomes of patients undergoing surgery for metastases are not affected by the region of the spine involved  (55).

### What About the Treatment of Asymptomatic Mets?

Early detection and treatment with radiation therapy of asymptomatic - radiological only - spinal cord compression helps preventing neurological deficits. In a study conducted on patients with metastatic prostate cancer, Venkitaraman et al. concluded that patients treated with radiation therapy for occult spinal cord compression detected on MRI had similar neurological deficit free survival as patients with no radiological spinal cord compression, highlighting the role of early radiotherapy in preventing neurological progression  (56).

### Surgical Complications

Tarawneh et al. reviewed the incidence of complications and unplanned re-operations after surgery for metastatic spinal tumors. Surgical site infection (6.5%) was the main reason for a re-operation in patients undergoing surgery for spinal metastases, followed by neurological deterioration (3%) and instrumentation failure (2%). Re-operation rate was estimated at 8%, with surgical site infection (28%) being the most common reason for revision surgery  (57).

When possible, minimally invasive techniques for decompression and stabilization seem to be the preferred method to surgically treat metastatic spine disease, with good outcomes  (58). For separation surgery, clinical outcomes were overall 1-year survival, 41-78%; recurrence rate, 4-22%; reoperation, 5%; and complications, 5-14%. For corpectomy, clinical outcomes were overall 1-year survival, 30-92%; reoperation, 1-50%; and recurrence rate, of 1-28%. Complications and reoperations with spinal instrumentation were 0-14% and 0-15%, respectively. Cement augmentation achieved pain reduction rates of 56-100%, neurologic improvement/stability 84-100%, and complication rates 6-56%. Laser achieved local tumor control rate of 71-82% at 1-year follow-up, reoperation rate of 15-31%, and complication rate of 5-26%  (58).

### Pain Control and Palliative Surgery

The postoperative median survival in spinal metastases cases is 38 weeks. In addition to the SINS, the primitive tumor type and the American Spinal Cord Injury Association (ASIA) score are considered significant prognostic survival factors  (59). A preoperative RT does not affect survival, nor did it increase the risk of wound infections  (60).

For biomechanically unstable fractures extending into the pedicle and/or articulation, posterolateral arthrodesis with percutaneous cement injection seemed to be a safe and effective therapeutic option for pain control  (61).

Mechanical radiculopathy in patients with spinal metastases represents a highly reliable surgical indication. Spinal decompression and fixation are effective treatments for pain palliation in such cases  (62).

### Advances in Radiotherapy

A lower SINS, indicating spinal stability, is associated with a complete pain response to radiotherapy. This supports the hypothesis that pain resulting from mechanical spinal instability responds less well to radiotherapy compared with pain from local tumor activity  (63, 64). However, no association could be determined between SINS and an overall pain response, which indicate that this referral tool is not yet optimal for prediction of treatment outcome  (65, 66). So although the SINS was initially developed for evaluating spinal instability in patients with spinal metastases, it is now commonly used to predict the occurrence of VCF after radiotherapy in patients with spinal metastases with moderate accuracy and substantial reliability  (67), the risk to develop new or worsening vertebral fractures being estimated around 20 %. Hence, SINS may also be used in predicting patients at high risk of spine stereotactic body radiation therapy (SBRT)-induced VCF  (68, 69). A higher spinal instability score increases the risk of RT failure and complications in patients with spinal metastases, regardless of the performance status, primitive tumor, and symptoms  (70). Baseline VCF and 18-24 Gy delivered in a single fraction were predictive of further collapse. Patients with oligometastatic disease may benefit most from such aggressive local therapy, given the prolonged survival observed  (71). 12-20% of lytic spinal lesions fractured at 1-year post-SBRT. A baseline fracture, spinal mal-alignment, and irradiation with ≥ 20 Gy/fx were positive predictive factors for VCF  (72).

In patients with poor prognosis presenting with cord compression secondary to metastatic epidural space invasion, multifraction radiotherapy (MFRT) has been considered as the standard of care (30 Gy in 10 fractions), but recent studies have proved that single fraction radiotherapy (SFRT) may also yield similar results in terms of functional and overall outcomes as well as pain control, and improvement of motor and bladder functions [**Table 3**; (74, 75)].

**Table 3:** Irradiation fractions of vertebral metastases [based on Thureau et al. (73) a French review concerning fractionations in radiotherapy of bone metastases].

Indications	Recommended fractioning	Other recommended fractioning	Remarks
Uncomplicated pain or neuropathic pain	30 Gy in 10 fractions	20 Gy in 5 fractions 8 Gy in 1 session 6 Gy in 1 session	Prefer fractioned regimens for spinal irradiation (risk of digestive toxicity)
Pain with vertebral fracture risk	30 Gy in 10 fractions	20 Gy in 5 fractions (exceptionally 8 Gy in 1 session)	Systematically rule out a surgical indication Achievement of cortical height is an important factor in assessing fracture risk
Post-operative RT	30 Gy in 10 fractions 20 Gy in 10 fractions	20 Gy in 5 fractions	
Inoperable spinal cord compression	30 Gy in 10 fractions	20 Gy in 5 fractions	
Re-irradiation	SRS 35 Gy in 5 fractions or 27 Gy in 3 fractions	20 Gy in 5 fractions (exceptionally 8 Gy in 1 session)	Take into account the dose already delivered, especially for the spinal cord
Oligometastatic cancer	SRS 35 Gy in 5 fractions or 27 Gy in 3 fractions		Take into account the patient's prognosis

### Palliative Radiotherapy and Pain Control

Pain in symptomatic vertebral metastases can be effectively controlled with radiation therapy. For the palliation of uncomplicated bone metastases, SFRT (8 Gy) and MSRT (30 Gy in 10 fractions) regimens provided similar pain control, and had equal toxicity risks  (76, 77). In such contexts, single dose should be preferred in palliative treatments  (77).In cases of reirradiation of spinal metastasis, SBRT can provide effective pain relief and neurologic improvement, with minimal toxicity  (78).

### Impact of SINS in Oncological Spine

Before the introduction of SINS in 2010, a consensus on the definition and assessment of neoplastic-related instability had been difficult to establish  (79). In reality, this scale has allowed a certain homogenization of the spinal neoplastic literature with more than 1500 articles being published on the matter since SINS first appearance  (44).

### Consequences in Medical and Surgical Training

SINS has good intra-observer and inter-observer reliability, hence its importance in guiding the management of patients with spinal metastases  (80). Hence, SINS appears as a reliable and valuable educational tool for spine fellows and residents learning to evaluate spinal instability  (81), as well as for spine surgeons, radiologists, and radiation oncologists.

### Limitation of the SINS

The main limitation of the SINS is that the majority of patients are generally classified as undetermined instability (7-12), a situation in which the experience of the specialist is still necessary for a subjective assessment of the conduct to be followed. One possibility could be to increase the cut-off value and revising the domains may improve the diagnostic performance to predict the risk of fracture of the SINS.

Furthermore, considering that the presence of neurological deficit would already indicate the need for specialized evaluation, the group of patients without deficit is the group where the SINS scale would have the greatest impact on screening patients at risk for instability.

In most of the cases there is also more than one spinal lesion. Eventually, the presence of lesions in adjacent vertebrae could increase the risk of mechanical complications. There are no modifiers for multiple spinal lesions in the SINS. It is known that the risk of instability increases in the junctional regions and mobile spine. Thus, lesions characterized as undetermined were more often found in these regions. Lesions located in the sacrum are rare and stable.

Kyphotic or scoliotic alignment disorders were also generally present in the cases of spines defined as unstable, indicating an important relationship between alignment and stability, and misalignement and instability. Stable spines generally did not present deformity in the sagittal or coronal planes.

Concerning the quality of the bone matrix, inter-observer assessment may have been inaccurate in judgment of the matrix quality and a source of error for the estimation of the SINS.

### Special Situations of Myeloma and Metastases of the Cranio-Cervical Junction

Similar to vertebral metastases, multiple myeloma and plasmacytoma of the spine could be responsible of VCF associated with significant morbidity and mortality due to severe back pain, spinal instability, increased risk of new fractures, neurologic dysfunction, and other physical symptoms  (13). In a 10-year analysis, Zadnik et al. report outcomes of surgical intervention for patients with indeterminate or gross spinal instability due to multiple myeloma with improved neurological function following surgery and low rates of instrumentation failure  (82). Recently, several factors were identified as predictive for vertebral collapse fractures in multiple myeloma patients: gender, International Staging System stage 2 and 3, and back pain  (83). Furthermore, lower Hounsfield Unit score, lytic lesions and abnormal alignment were risk factors for the development of VCF  (83).

Metastasis to cranio-cervical junction may result in instability manifesting by disabling pain, cranial nerve dysfunction, paralysis, or even death. Stabilization is required to prevent complications. Nonoperative treatment modalities are ineffective in providing stability and adequate pain relief. C2 body is the most common site of metastasis. Occipito-cervical fusion for unstable upper cervical metastasis offers a good palliative treatment for pain relief and improved quality of life  (84).

### New Predictive Scores

SINS served as the basis for the development of new scores as the treatment strategy algorithm of Paton et al. (85) (LMNOP), that evaluates the number of spinal Levels involved, the Location of disease in the spine (L), Mechanical instability (M), Neurology (N), Oncology (O), Patient fitness, Prognosis and response to Prior therapy (P). It helps taking the best decision in each particular patient. LMNOP is the first systematic approach to spinal metastasis that incorporates SINS. It is easy to use, it addresses clinical factors that weren’t previously mentioned in other scoring systems, and it is adaptable to changes in technology  (86). The Oswestry Spinal Risk Index (OSRI) is another simple score complementary to the SINS that can predict life expectancy accurately in patients presenting with spinal metastases  (87). It is helpful in making difficult clinical decisions without the delay of extensive investigations.

### Biomarkers

Most of the research has been focused on the pathophysiology and treatment of bone metastases, with only few studies investigating potential predictors of risk for bone metastases development  (88). There is thus a need for biomarkers, which will predict the risk of spreading to bone within the major bone metastatic cancers (breast, prostate, lung and renal cell carcinoma). The application of molecular profiling techniques, together with animal model systems and engineered cell-lines has enabled the identification of a series of potential bone-metastasis biomarker molecules predictive of bone metastasis risk. Some of these biomarker candidates have been validated within patient-derived samples providing a step towards clinical utility. Recent developments in multiplex biomarker quantification technologies accelerated things even more, allowing to identify novel drug targets within cancer spread to bone (89).

### The Future: Machine Learning Algorithms and Artificial Intelligence

In 2017, the International Spine Oncology Consortium reported an integrated multidisciplinary algorithm for the management of spinal metastases  (90). Thus, the Skeletal Oncology Research Group (SORG) machine-learning algorithms can preoperatively predict the overall survival in spinal metastatic disease  (91). Hence, machine learning algorithms are promising tools and will most likely become essential for the management of spinal metastases in the next few years. Artificial intelligence becomes useful in spinal lesion assessment and can now discriminate between benign, primary malignant or metastases  (92). Moreover, some machine learning algorithms can already predict bone metastasis in patients with newly diagnosed thyroid cancer for example  (93). Artificial intelligence outlined better survival after surgery for spinal metastases in comparison to traditional risk scores like SINS or Tokuhashi  (43). Other machine learning algorithms can predict VCF at one year after SRT with excellent sensitivity and specificity, outperforming models developed from clinical features or components of the SINS alone  (94). So, we are now on the verge of a real technological revolution which will literally turn the care of patients with spinal metastases upside down over the next few years. However, these tools still need to be tested on very large cohorts of patients to be validated. In the meantime, scores like SINS remain more up to date than ever.

## Conclusions

An improved understanding of tumor biology, the ability to target tumor drivers, and the ability to harness the immune system have dramatically improved the expected survival of patients diagnosed with cancer. However, many patients continue to develop spine metastases that require local treatment with RT and surgery. Fortunately, the evolution of radiation delivery and operative techniques enables durable tumor control with a decreased risk of treatment-related toxicity and a greater emphasis on restoration of quality of life and daily function. The SINS score was introduced ten years ago as a tool that helps clinicians and specialists (radiologists, oncologists, radiotherapists, spine surgeons, and trainees) to determine whether instability is present and when surgery may be indicated. Among patients who still require surgery for decompression of the spinal cord or column stabilization, minimal access approaches and targeted tumor excision and ablation techniques reduce the surgical risk and accelerate postoperative recovery. In other cases, stereotactic radiotherapy allows delivery of ablative radiation doses to the majority of spinal metastases, even in the cases of palliative surgery. Hence, SINS had significantly contributed to synergic interdisciplinary collaboration among clinicians in the interest of patients with spinal metastases.

## Author Contributions

NS wrote the first draft of the manuscript. JF, BT, MC, SA, and GA wrote sections of the manuscript. All authors contributed to manuscript revision, read, and approved the submitted version.

## Conflict of Interest

The authors declare that the research was conducted in the absence of any commercial or financial relationships that could be construed as a potential conflict of interest.

## Publisher’s Note

All claims expressed in this article are solely those of the authors and do not necessarily represent those of their affiliated organizations, or those of the publisher, the editors and the reviewers. Any product that may be evaluated in this article, or claim that may be made by its manufacturer, is not guaranteed or endorsed by the publisher.

## Abbreviations

ASA, American Society of Anesthesiologists; ASIA, American Spinal Cord Injury Association; CT, Computed Tomography; KPS, Karnofsky Performance Status; LMNOP, Location/Mechanical instability/Neurology/Oncology/Patient fitness, Prognosis and response to Prior therapy; MFRT, Multifraction Radiotherapy; MRI, Magnetic Resonance Imagery; OSRI, Oswestry Spinal Risk Index; ENT, Ear-Nose-Throat; SBRT, Stereotactic body Radiation Therapy; SFRT, Stereotactic Functional Radiotherapy; SINS, Spine Instability Neoplastic Score; SOSG, Spine Oncology Study Group; SRS, stereotactical radiosurgery; SRT, stereotactical radiotherapy; VAS, Visual Analogic Scale; VCF, vertebral compression fracture; WHO, World Health Organization.
